# Concurrent sternal and pulmonary foci of Langerhans cell histiocytosis

**DOI:** 10.4103/1817-1737.65037

**Published:** 2010

**Authors:** Young Hwan Koh, Hyeon Jong Moon, Byung-Su Kim, Dae Hee Han

**Affiliations:** *Department of Radiology, Seoul St. Mary's Hospital, The Catholic University of Korea, 505, Banpo-dong, Seocho-gu, Seoul – 137 701, Korea. E-mail: lepolder@gmail.com*

Sir,

We recently came across a case of concurrent sternal and pulmonary langerhans cell histiocytosis (PLCH), who was successfully diagnosed by a careful review of high-resolution computed tomography (HRCT) and using ultrasound (US) guidance for tissue diagnosis. PLCH is part of the spectrum of the disorder manifesting as an interstitial lung disease[1] with an uncommon (4–20%) association of bone lesions in adult patients.[2] The sternum is a rare site of LCH involvement[3] and there has been no report of simultaneous involvement of lung and sternum in LCH. The patient, a 32-year-old male, presented with a 40-day history of anterior chest pain and swelling. US showed a low-echoic mass [Figure 1a] in the sternum with soft-tissue extension and cortical disruption, prompting the possibility of tuberculosis and metastasis. We noticed bilateral interstitial infiltration [Figure 1b] on the radiograph and the patient underwent HRCT. HRCT revealed bilateral diffuse infiltrates sparing the costophrenic angle, composed of well-defined small nodules with occasional cavitation [Figure 1c]. Under the strong impression of PLCH with concomitant sternal involvement, US-guided biopsy of the sternal lesion was done with no associated complications. The histologic findings were consistent with LCH [Figure 1d], with immnohistochemistry revealing the following results: focal positive for S100; focal positive for CD3 in T cells; focal positive for L26 in B cells; negative for CD30 and CD15. Treatment with vinblastine and prednisone with mercaptopurine, brought a rapid symptom relief.

**Figure 1a:**
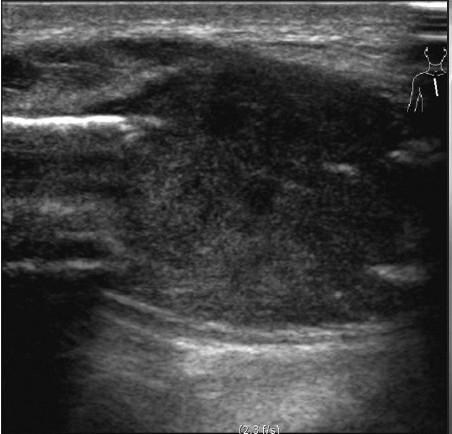
A 32-year-old male with concurrent sternal and pulmonary LCH. (a) Sagittal ultrasonogram of the sternum shows a heterogeneous low-echoic mass causing cortical disruption and soft-tissue extension.

**Figure 1b:**
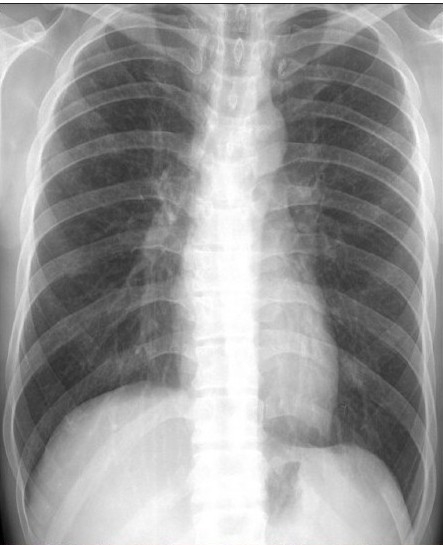
Posteroanterior radiograph of the chest shows fine reticular opacities in the upper and middle zones of both lung fields.

**Figure 1c:**
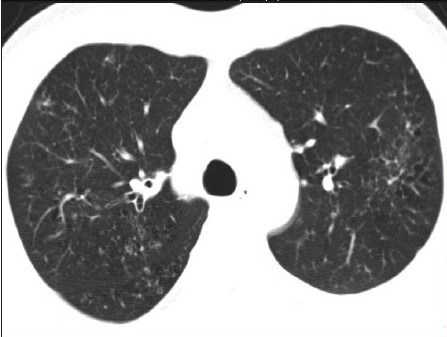
HRCT shows well-defined small nodules with occasional cavitation.

**Figure 1d:**
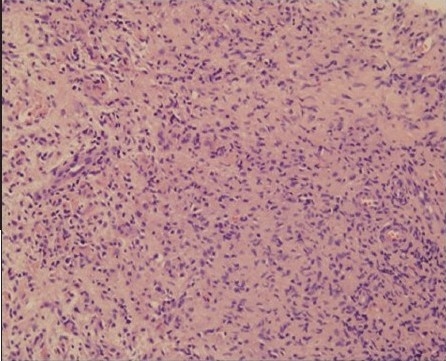
Histologic features (hematoxylin and eosin, ×200) are histologic features characteristic of LCH.

Although sternal involvement of LCH is rare and manifests with nonspecific physical and radiologic signs, PLCH presents with specific HRCT signs. Therefore, chest radiograph of a patient with a painful sternal mass should be scrutinized for any interstitial infiltrates that may lead to a specific imaging diagnosis, which was PLCH in this case.

To date, sternal LCH has been diagnosed using various surgical techniques. However, tissue diagnosis can be done percutaneously using US guidance as in this case, reducing the morbidity and the length of hospital stay.
